# Evidence for Air Movement Signals in the Agonistic Behaviour of a Nocturnal Arachnid (Order Amblypygi)

**DOI:** 10.1371/journal.pone.0022473

**Published:** 2011-08-10

**Authors:** Roger D. Santer, Eileen A. Hebets

**Affiliations:** 1 School of Biological Sciences, University of Nebraska Lincoln, Lincoln, Nebraska, United States of America; 2 Institute of Biological, Environmental, and Rural Sciences, Aberystwyth University, Aberystwyth, United Kingdom; University of Arizona, United States of America

## Abstract

Many arthropods possess filiform hair sensilla (termed trichobothria in arachnids), which are extremely sensitive detectors of medium particle displacement. Electrophysiological evidence in some taxa suggests that these sensilla can detect air particle displacements resulting from intraspecific communication signals. However, it has not yet been shown for any species that the air particle displacements detected by the filiform hairs are themselves perceived as a ‘signal’ (i.e. that individuals make behavioural decisions based upon the responses of these organs to the displays of conspecifics). We investigate the agonistic behaviour of the whip spider *Phrynus marginemaculatus* and the role of its trichobothria in receiving agonistic signals. Whip spiders have extremely elongated ‘antenniform’ first legs, which they vibrate close to their opponents during agonistic interactions, inducing air movements that excite their opponents' trichobothria. We find that ablation of the trichobothria causes significant increases in: (I) contest duration, and (II) the probability of contest escalation past aggressive displays to physical fighting. Therefore, in the absence of air movement-sensitive sensilla, contest assessment is impaired. This suggests that whip spiders exploit true air movement signals during agonistic interactions, and that these are received by the trichobothria. Furthermore, these results indicate that, in whip spiders, such signals help mitigate the cost of agonistic interaction.

## Introduction

Many insects, arachnids and crustaceans possess arrays of filiform hair sensilla (termed trichobothria in arachnids) which are sensory structures that detect air (or water) particle displacements [1], [2]. In terrestrial arthropods, filiform hairs are extremely sensitive to air currents inadvertently induced by movement, and in this way they enable a variety of species to detect and evade predators, or capture prey (e.g. [3], [4], [5], [6]). Whether or not these hairs also operate as receivers of air particle displacements advertently induced as intraspecific signals is less well understood, but this is an important research question, both because the broad distribution of filiform hairs across many arthropod taxa could mean that such a form of communication is extremely prevalent, and because these may be rewarding systems for investigating hypotheses of signal evolution [7].

A role for filiform hair sensilla in receiving intraspecific air movement signals has so far been proposed for a cave cricket and a whip spider (and hypothesised through exclusion of other signalling modalities for a wolf spider) [7], [8], [9]. The African cave cricket *Phaeophilacris spectrum* lacks stridulatory organs, but males make silent wing flicks during courtship and aggression that induce females to be less sensitive to disturbing tactile or vibratory stimuli [10], and induce air currents that excite the filiform hairs of females (e.g. [8]). The whip spider *Phrynus marginemaculatus* (Arachnida, Amblypygi) has extremely elongated ‘antenniform’ first legs. During agonistic contests, a whip spider extends one antenniform leg straight, and rapidly vibrates it close to, but rarely making contact with, its opponent [11], [12]. These antenniform leg vibration (ALV) displays induce air particle movements that excite the walking leg trichobothria of the receiver [7]. Therefore, in both cases, the display movement of the signaller appears to be a communication signal, and it elicits air particle displacements that excite filiform hairs of the receiver. However, it has not been shown in these, or any species, that air movements detected by the filiform hairs (rather than stimuli emanating from the display in other modalities) are actually perceived as signals by the receiver and influence behavioural decisions. Only by examining the link between stimulus perception and an altered probability pattern of behaviour (see [13]), can the prevalence of communication through air particle displacement be assessed.

In the case of *Phrynus marginemaculatus* whip spiders, agonistic contests consist of a stereotyped sequence of aggressive displays that escalates until one competitor retreats (e.g. [11], [12], [14]). When contests begin, each whip spider orients to face its opponent and reaches forwards with both its (partially flexed) antenniform legs to gently and slowly touch various areas of its opponent's body (‘probing’) [11], [12], [14]. This behaviour gradually becomes more intense, with faster probing movements and increasingly jerky, sometimes quivering movements of the still partially-flexed antenniform legs during and between probing contacts with the opponent (Santer and Hebets, pers. obs.). Retreat of one whip spider will sometimes occur, but commonly (in all contests between naïve males and 82% of contests between males that had previous contest experience), probing movements become interspersed with more discrete, easily-identifiable aggressive displays including more vigorous vibration of the straightened antenniform legs (seldom making contact with the opponent), elevated body posture, and pedipalp opening displays [11]. ALV is among the most striking of these displays, and is performed by both individuals in 91% of contests between naïve males and 64% of contests between experienced males [7], [11]. Normally one whip spider will retreat following aggressive displays, but 27% of contests between naïve males and 9% of contests between experienced males escalate to a final phase in which whip spiders physically fight using their spiny pedipalps [11]. Relative ALV display duration is a predictor of contest outcome, even in complete darkness [7], [11]. ALV is directed over the opponent's walking legs, which bear the vast majority of trichobothria (outside of the walking legs, seven additional trichobothria are found on the tibia of the antenniform leg [15]), and electrophysiological experiments with simulated ALV displays show that they induce air particle displacements that excite the mechanoreceptors underlying these walking leg trichobothria [7]. ALV is, therefore, a strong candidate for a signal operating in the air particle displacement modality, but it may be that other less discrete and easily identifiable, jerky or quivering movements of the antenniform legs also operate in this way.

To test whether *P. marginemaculatus* perceives air particle displacements as signals during agonistic contests and uses them to guide its behavioural decisions, we compare the contest behaviour of whip spiders with intact trichobothria (male and female control groups), with that of whip spiders with all walking leg trichobothria ablated (male and female TA [trichobothria ablated], groups). Whip spiders with walking leg trichobothria ablated would have limited ability to detect air particle displacements, and thus would not have access to putative air particle displacement signals. Therefore, we predict that if air particle displacements from ALV or other antenniform leg movements truly operate as signals: (I) animals in the TA groups should take longer to gather information and assess their opponents, leading to an increase in the time necessary to make a behavioural decision to retreat or escalate that would be evident as an increase in overall contest duration; and (II) contests between animals in the TA groups would be more likely to escalate past the un-assessable air movement signals to a phase of physical fighting using the pedipalps, because the air movement signals could not motivate a decision to retreat. Since such contest parameters are also proxies for the cost of agonistic competition, such findings would also provide direct evidence that agonistic signals mitigate the costs of agonistic interaction in whip spiders (e.g. [16]).

## Results

### General contest behaviour

At the start of trials, whip spiders typically circled the arena. Contests usually began when whip spiders first physically touched one another (normally with their antenniform legs), but sometimes circling would continue after such contact was briefly made, and the contest start would be delayed. No significant difference was found in the latency from the start of a trial to the start of a contest between the control and TA groups, either for males (Wilcoxon-Mann-Whitney (WMW) test, W = 33.5, n1 = n2 = 6, p = 0.422, power = 0.29), or for females (WMW test, W = 44.0, n1 = n2 = 6, p = 0.471, power = 0.14) (table 1). During this latency period, circling exploration of the arena was occasionally punctuated with bouts of grooming behaviour. No significant difference was found in the number of grooming bouts per individual per minute of latency period between control and TA groups, either for males (WMW test, W = 37.0, n1 = n2 = 6, p = 0.753, power = 0.06), or for females (WMW test, W = 37.5, n1 = n2 = 6, p = 0.864, power = 0.06) (table 1).

**Table 1:** Summary data for selected measurements of the agonistic contests of pairs of male or female whip spiders with trichobothria intact (controls, C group), or trichobothria ablated (TA group).

		Latency	Pre-contest grooming bouts	In contest grooming bouts	Winner ALV duration	Loser ALV duration
		(s)	(/individual/min)	(/individual/min)	(s)	(s)
**Males**						
*C group*	*mean*	394.5	0.04	0.00	111.0	98.8
	*median*	185.5	0.00	0.00	104.0	84.5
	*SD*	399.4	0.07	0.00	113.8	101.8
*TA group*	*mean*	132.5	0.03	0.02	247.0	221.3
	*median*	152.5	0.00	0.00	234.0	135.5
	*SD*	52.0	0.07	0.05	214.9	264.1
**Females**						
*C group*	*mean*	317.3	0.12	0.03	51.7	46.2
	*median*	146.5	0.07	0.00	41.5	38.0
	*SD*	483.5	0.14	0.06	45.3	41.6
*TA group*	*mean*	717.0	0.09	0.02	62.2	43.3
	*median*	362.0	0.04	0.01	24.0	24.0
	*SD*	893.7	0.15	0.03	82.2	52.0

Each group comprises six agonistic contests. SD, standard deviation; ALV, antenniform leg vibration.

During contests, both control and TA group whip spiders performed the typical range of contest behaviours: antenniform leg probing; jerky and quivering movements of the antenniform legs including antenniform leg vibration (ALV); pedipalp opening displays; and physical fighting using the pedipalps [7], [11], [12], [14]. Commonly within the TA group (but less commonly within the control group), ‘lulls’ in contest behaviour were observed, during which the intensity of the contest appeared to decrease momentarily and whip spiders used their antenniform legs to probe the area of arena surrounding them. Occasionally, bouts of grooming by one or both individuals occurred during these lulls. Grooming behaviours were seen during no male and one female control group contest, versus in two male and three female TA group contests (from a total of six contests in each treatment×sex group). The number of grooming bouts per individual per minute could not be compared between male control and TA groups (using WMW tests; all values for the control group were zero), but no significant difference was found between female control and TA groups (WMW test, W = 43.5, n1 = n2 = 6, p = 0.446, power = 0.06) (table 1).

### ALV display occurrence and duration

Both whip spiders performed ALV in five male, and five female, control group contests. In each remaining male and female control group contest one whip spider performed ALV. Both whip spiders performed ALV in all male, and four female, TA group contests. In the remaining two female TA group contests neither whip spider performed ALV. However, jerky or quivering antenniform leg movements other than ALV were evident in all contests.

No significant difference was found in the total ALV duration of the winning whip spider between control and TA groups, either for males (WMW test, W = 46.0, n1 = n2 = 6, p = 0.298, power = 0.23) or for females (WMW test, W = 35.0, n1 = n2 = 6, p = 0.573, power = 0.06) (table 1). Likewise, no significant difference was found in the total ALV duration of the losing whip spider between control and TA groups, either for males (WMW test, W = 45.0, n1 = n2 = 6, p = 0.379, power = 0.15), or for females (WMW test, W = 36.0, n1 = n2 = 6, p = 0.688, power = 0.05) (table 1).

### Overall contest duration

**Figure 1:**
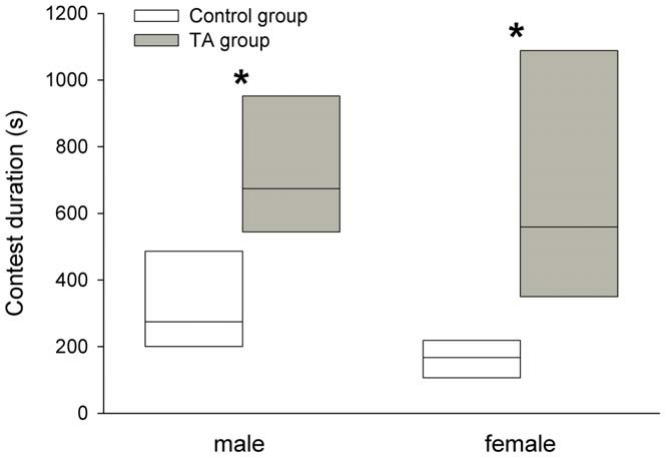
Duration of agonistic contests between whip spiders with trichobothria intact or trichobothria ablated. Boxes for the trichobothria intact (control group, white) and trichobothria ablated (TA group, grey) groups each show the median, 25^th^ and 75^th^ percentiles for the contest durations of 6 contests between naïve pairs of whip spiders. Asterisks indicate significant differences (p<0.05) as identified by WMW tests (see text).

There was a significant difference in total contest duration between the control and TA groups, both for male (WMW test, W = 52.0, n1 = n2 = 6, p = 0.045, power = 0.61), and for female whip spiders (WMW test, W = 55.0, n1 = n2 = 6, p = 0.013, power = 0.66) (fig. 1).

### Contest escalation

**Figure 2:**
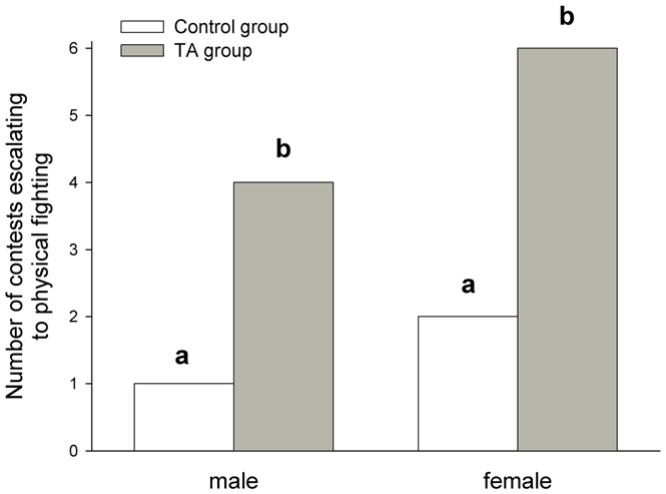
Escalation of agonistic contests between whip spiders with trichobothria intact or trichobothria ablated. Bars show the number of agonistic contests escalating to physical fighting (defined as one or both whip spiders making physical contact with their opponent using their pedipalps), in the trichobothria intact (control group, white) and trichobothria ablated (TA group, grey) groups. Each experimental group contained 6 contests between naïve pairs of whip spiders. Bars labelled ‘a’ and ‘b’ differ significantly at p<0.01, as determined by a log-linear analysis of the three-way contingency table treatment×sex×physical fighting occurrence (see text).

One male and two female control group contests, versus four male and six female TA group contests (from a total of six contests in each treatment×sex group), escalated to physical fighting (defined as one or both whip spiders making physical contact with their opponent using their pedipalps). We performed a log-linear analysis of the three-way contingency table of treatment group (T)×sex (S)×physical fighting occurrence (F). This identified significant differences in contest escalation to physical fighting between the control and TA groups (G^2^[TSF] = 12.42, df[TSF] = 4, p = 0.015; G^2^[TF] = 8.79, df[TF] = 1, p = 0.003; G^2^[TSF-TS-SF] = 10.89, df[TSF-TS-SF] = 2, p = 0.004; G^2^[TSF-TF] = 3.63, df[TSF-TF] = 3, p = 0.304) (fig. 2).

In the male and female control groups, and the male TA group, physical fighting usually began in the final stages of contests. The proportion of total contest duration that had elapsed before the first occurrence of physical fighting in each group was: male control group, 0.76 (n = 1); female control group, 0.91 (0.91)±0.19 (n = 2); male TA group, 0.83 (0.99)±0.32 (n = 4) [mean (median) ± SD]. In the female TA group, physical fighting often first occurred earlier: the mean proportion of total contest duration that had elapsed before the first bout of physical fighting was 0.15 (0.15)±0.13 (n = 6). This was often prior to the first clearly identifiable ALV display, but was always preceded by jerky or quivering antenniform leg movements by one or both whip spiders.

## Discussion

Whip spider agonistic contests are an escalating sequence of displays that can culminate in physical fighting using the pedipalps if competitors are equally matched [11], [12], [14]. It has been suggested that air movement cues generated by the ALV display and received by the trichobothria serve as agonistic signals [7]. If these or other air movements are used in communication, preventing their reception would be predicted to cause: (I) an increase in contest duration, and (II) an increased frequency of escalation past aggressive displays to physical fighting using the pedipalps. These effects are the predicted results of a receiver's inability to make an assessment of its opponent upon which to base a behavioural decision. Here we find that ablation of the walking leg trichobothria (which prevents the detection of air movement cues) results in exactly these effects on whip spider contest behaviour. Therefore, our results suggest that air movements are perceived as signals by whip spiders, providing the first behavioural evidence that air movements received by filiform hairs can truly operate as signals in any arthropod. Since filiform hairs are widely distributed among arthropods, this kind of air movement-based communication could be extremely widespread [7]. Furthermore, because contest duration and escalation level are correlates of fighting cost, our results provide useful evidence that signalling mitigates the cost of agonistic competition in this species [16].

Whip spider trichobothria serve many behavioural functions, including locating moving prey, and triggering startle responses (e.g. [17], [18]). However, we did not see obvious affects of trichobothria ablation on the latency to the start of a contest, or on occurrences and rates of grooming behaviour as whip spiders explored their arena pre-contest. Furthermore, we observed the usual stereotyped display behaviours during agonistic contests, and there were no obvious differences in ALV display duration (a behaviourally important parameter, [7], [11]), as a result of our manipulation. However, it should be noted that our statistics had limited power with which to test these observations.

Consistent with our hypothesis that the trichobothria receive air movement signals during agonistic contests, we saw unusual pauses in display behaviours during TA group contests without retreat of either competitor. It seems likely that these pauses occurred because their inability to receive air movement signals meant that whip spiders were unable to assess their opponents and make the behavioural decision to retreat or escalate the contest. Also consistent with our hypothesis, pauses in display behaviours were often associated with bouts of grooming behaviour (although the occurrence of these did not differ significantly between control and TA group contests). Animals sometimes groom during agonistic interactions and this is interpreted as a displacement activity (e.g. [19]). However, in our study it is also possible that whip spiders were attempting to recover the function of their trichobothria by grooming, in order to gain the information necessary for assessment of their opponent.

The effects of the TA manipulation on contest duration and escalation were consistent in males and females, but some aspects of female contests were unusual. In two female TA group contests, ALV displays did not occur, and in general TA group females tended to engage in physical fighting early in a contest, before ALV had occurred. ALV is the only whip spider agonistic signal so far proposed to operate through air particle displacement [7], but a variety of less discrete, jerky or quivering movements of one or both antenniform legs occur throughout agonistic contests that might also function in this way. Such movements of the antenniform legs were observed in all contests, and always preceded physical fighting in female TA group contests. Thus, potential air movement signals were performed in all contests and before escalation to physical fighting, even if ALV displays were not. Furthermore, ‘early’ physical fighting in female TA group contests did not lead to contest resolution and there was always a de-escalation to subsequent phases of probing and/or aggressive signalling (often including ALV). There was, therefore, clearly still an attempt to use further aggressive signalling for contest resolution. As such, increased contest duration and escalation in the female TA group might still be explained by their compromised abilities to perceive and assess air movement signals resulting from ALV and other antenniform leg movements.

Previous studies have shown that contests between females are typically shorter than those between males, that ALV is less likely to occur, and that physical fighting using the pedipalps is slightly more likely to occur [11]. It has also been suggested that there may have been less selection for ritualisation in female whip spider contests [11]. In other species (a jumping spider and a cichlid fish) it is suggested that similar variation between male-male, and female-female contests results from a ‘desperado effect’ in females, whereby decisions to persist in a contest relate to their assessment of resource payoff value rather than opponent resource holding potential [20], [21]. Such factors may also have contributed to variation in contest behaviour between males and females, and the differences between male and female agonistic signalling, and its evolution, are certainly worthy of attention in future work.

Although our study was primarily aimed at testing the hypothesis that air movements have a signalling function during whip spider interactions, our findings also provide good evidence that such signals mitigate the costs of aggressive interaction. This is because contest escalation and duration are expected to covary with energy expenditure, meaning that without signals, agonistic interactions are more costly to competitors. Although this is a key assumption of models of agonistic communication, direct evidence for it is sparse (see [16]). Logue et al (2010) demonstrated that agonistic contests were more costly between field crickets (*Teleogryllus oceanicus*) that could not signal by producing sound (either as a result of an evolved signal loss, or an experimental manipulation) [16]. Additionally, Rillich et al (2007) undertook sensory ablation experiments on the field cricket *Gryllus bimaculatus*, and found that contests between experimentally blinded male crickets had higher levels of escalation than contests between crickets that could see normally [22]. Both of these studies, and our own, provide good evidence that agonistic contests are more costly when competitors cannot send and receive signals.

Our findings provide behavioural evidence that whip spiders perceive air movement signals detected by their trichobothria during agonistic contests and make behavioural decisions based upon them. They also provide evidence that these signals help to mitigate the costs of aggressive contests. Given the widespread occurrence of filiform hairs among arthropods, the occurrence and evolution of this form of communication is an important subject for future research.

## Materials and Methods

### Ethical statement

Our experiments comply with the current animal protection laws of the USA, which do not require ethical approval for experiments on arachnids. Nonetheless, care was taken not to over-collect whip spiders, and all possible efforts were made to minimise distress caused to them during experimentation. No whip spiders died as a result of experimentation, and even those with trichobothria removed are able to regenerate these sensilla at their next moult.

Experiments were performed on 16 adult *Phrynus marginemaculatus* (8 male and 8 female), collected from Big Pine Key, Florida, USA, in August 2006. This sample size was restricted by several important factors: this species of whip spider is difficult to find, and adults could only be collected in limited numbers; whip spiders are long-lived, meaning that it was not feasible to collect juveniles and mature them in the laboratory for experimentation; and because their ecology is poorly understood, it was crucial not to over-collect them. Whip spiders were individually housed in the laboratory (see [7]).

The eight whip spiders of each sex were divided into control and trichobothria ablated (TA) groups. TA group whip spiders were anaesthetized on ice and all walking leg trichobothria removed by gentle rubbing over each trichobothrium socket using a hypodermic needle, breaking the trichobothrium off at its base (e.g. [23]). The majority of trichobothria are located on the walking legs [14], but there are a number of short trichobothria on the tibia of the antenniform leg [15], that were not removed in our experiments. These antenniform leg trichobothria are some distance from the body and usual location of the ALV display [7]. Control group whip spiders were identically anesthetized and adjacent areas of cuticle without trichobothria were gently rubbed. Whip spiders were returned to their individual cages and fed one recently-killed cricket, preventing TA whip spiders being nutritionally disadvantaged since trichobothria are used to locate moving prey [24].

Agonistic contests were staged between naïve pairs of whip spiders within each treatment×sex group at 2 day intervals. Because treatment×sex groups contained 4 individuals, 6 contests were possible for each (3 contests/individual). Contests were staged during the dark phase of the whip spiders' light cycle (their normal active phase) in a 20 cm diameter arena with 5 cm high acetate walls. Illumination was from an IR LED array and other lighting was excluded; contests were filmed from above using a DCR-HC65 camcorder with IR capability (Sony Electronics Inc., USA). For each contest, one whip spider was introduced on each side of the arena under an upturned glass vial. After a brief acclimatization period, vials were removed and whip spiders released. Whip spiders do not normally aggregate in same-sex groups (e.g. [25]), and sometimes try to avoid one another in an experimental arena. Because we were interested in air movement signals, we allowed repeated interactions until the probing phase of contest behaviour began. Following resolution of the contest, whip spiders were returned to their home cages, fed one dead cricket, and allowed a rest day before their next contest.

Video recordings were digitized using Windows Movie Maker (Microsoft Corporation, USA) and played back using Windows Media Player (Microsoft Corporation, USA) on a standard PC. For the purposes of analysis, we defined the timing of a contest start as the moment that a whip spider first oriented towards a clearly aggressive opponent, without either animal turning or walking away immediately following this reorientation. We defined contest resolution as one individual (the losing whip spider) retreating through a radius of three body lengths from its opponent's (the winning whip spider's) chelicerae, with continuing motion away from the winner [11]. On this basis we measured overall contest durations (seconds). We also recorded whether or not each contest escalated to physical fighting (defined as one or both whip spiders making physical contact with their opponent using their pedipalps).

Each contest involved the unique interaction of a pair of naïve whip spiders and is, therefore, considered as an independent observation for analysis. Since sample sizes were small, nonparametric Wilcoxon-Mann-Whitney (WMW) tests were used to test for significant differences in contest measurements between control and TA groups, and these tests were always adjusted for ties where applicable. The proportion of contests escalating to physical fighting were arranged as a three-way contingency table of treatment group (T)×sex (S)×physical fighting occurrence (F) and a log-linear analysis was performed. Statistical analyses were carried out using Minitab v.14 (Minitab Inc., USA), and the VasserStats online calculator [26]. It should be noted that, due to the unavoidably small sample size, the statistical power of WMW tests (calculated using G*Power 3.1.3 [27]), would have been adequate to detect only large effect sizes (d) at α 0.05 (statistical power: 0.99 for d = 3.0; 0.86 for d = 2.0; 0.62 for d = 1.5; 0.33 for d = 1.0; 0.12 for d = 0.5), and was often low when calculated from sample effect sizes (results). For completeness, we state instances in which statistics could not refute null hypotheses, but we remind the reader to keep in mind limitations of statistical power. We provide descriptive statistics for non-significant results (table 1), and plot significant results (fig. 1 and 2), so that our data may be directly assessed.

Finally, data relating to multiple pair-wise interactions between a fixed number of individuals have sometimes been analysed using Mantel tests which do not assume independence of the data points (e.g. [20]). However, it has been argued that these tests should only be applied where hypotheses are formulated in terms of distances (e.g. [28]). We performed Mantel tests on our data using Mantel.xla [29] and these were in general agreement with the statistical tests reported in this manuscript. However, we do not report Mantel test results due to doubts over their applicability.
